# Use of Hemagglutinin Stem Probes Demonstrate Prevalence of Broadly Reactive Group 1 Influenza Antibodies in Human Sera

**DOI:** 10.1038/s41598-018-26538-7

**Published:** 2018-06-05

**Authors:** Hadi M. Yassine, Patrick M. McTamney, Jeffery C. Boyington, Tracy J. Ruckwardt, Michelle C. Crank, Maria K. Smatti, Julie E. Ledgerwood, Barney S. Graham

**Affiliations:** 1 Qatar University Biomedical Research Center, Doha, 2713 Qatar; 2 Medimmune, Gaithersburg, MD 20878 USA; 3 Vaccine Research Center, National Institute of Allergy and Infectious Diseases, Bethesda, MD 20892 USA

## Abstract

A better understanding of the seroprevalence and specificity of influenza HA stem-directed broadly neutralizing antibodies (bNAbs) in the human population could significantly inform influenza vaccine design efforts. Here, we utilized probes comprising headless, HA stabilized stem (SS) to determine the prevalence, binding and neutralization breadth of antibodies directed to HA stem-epitope in a cross-sectional analysis of the general population. Five group-1 HA SS probes, representing five subtypes, were chosen for this analyses. Eighty-four percent of samples analyzed had specific reactivity to at least one probe, with approximately 60% of the samples reactive to H1 probes, and up to 45% reactive to each of the non-circulating subtypes. Thirty percent of analyzed sera had cross-reactivity to at least four of five probes and this reactivity could be blocked by competing with F10 bNAb. Binding cross-reactivity in sera samples significantly correlated with frequency of H1^+^H5^+^ cross-reactive B cells. Interestingly, only 33% of the cross-reactive sera neutralized both H1N1 and H5N1 pseudoviruses. Cross-reactive and neutralizing antibodies were more prevalent in individuals >50 years of age. Our data demonstrate the need to use multiple HA-stem probes to assess for broadly reactive antibodies. Further, a universal vaccine could be designed to boost pre-existing B-cells expressing stem-directed bNAbs.

## Introduction

Annual influenza epidemics affect up to 15% of the world population and cause about 500,000 annual deaths globally. Influenza viruses also periodically cause pandemics, the most recent being in 2009 caused by swine-origin H1N1 virus^1^. The antibody response to current influenza vaccines primarily target the head region of the hemagglutinin (HA) glycoprotein, which is subject to constant antigenic drift, necessitating annual updates of influenza vaccines^2^.

Antibodies with broad specificity have been isolated from humans, including those that bind conserved epitopes within the stem region of HA^3–7^. HA stem-specific antibodies can have cross-subtype specificity within groups (e.g. CR6261- and F10-like for group 1^3^ or CR8020 for group 2^7^) or cross-group specificity (e.g. FI6, CT149 and CR9114^5,6^). Those that target group 1 viruses have been frequently isolated from human subjects vaccinated or infected with influenza virus^8–10^. Interestingly, more than two-thirds of such antibodies are derived from the heavy chain *VH1-69* gene family, which requires little maturation to achieve broad reactivity^11^.

The ability to elicit broadly cross-reactive antibodies against the conserved stem of HA could be the basis for an influenza vaccine capable of providing protection against various antigenically distinct or drifted influenza strains^2^. In principle, an HA stem-targeting, broad specificity influenza vaccine would not require annual updates, and would induce near universal immunity against diverse influenza viruses. For example, it has been shown that vaccination with H1-based HA-stabilized stem (SS) nanoparticles, that have the variable HA head region removed, elicit broadly cross-reactive antibodies and provides protection in mice and ferrets against lethal heterosubtypic H5N1 influenza virus challenge despite the absence of detectable H5N1 neutralizing activity *in vitro*. Further, passive transfer of immunoglobulin from H1 HA SS nanoparticles–immunized mice to naive mice resulted in full protection from lethal H5N1 challenge, indicating that HA stem–specific antibodies protect against diverse group 1 influenza subtypes in animal models^12^. Similar vaccination strategies have been reported against group 2 influenza A subtypes as well^13^.

Accordingly, reliable methods to detect and assay for broadly reactive stem-specific antibodies will be needed to determine their prevalence in the human population, and also to assess the efficacy of next-generation influenza vaccines. Although previous studies have interrogated the prevalence of broadly-reactive stem-directed antibodies in humans using various methods including competition assays, chimeric HA, or phage display methods^14–20^, none of these studies used structurally-defined stem-only probes to measure binding and stem-specific neutralizing activity in human sera. Here, we present a new set of structurally-defined^12^ stabilized-stem probes (seasonal and pandemic H1, H2, H5, and H9) to determine the prevalence, frequency, breadth and specificity of broadly reactive antibodies in human sera. Analysis of 202 human sera samples revealed a wide prevalence of broadly-reactive antibodies to multiple group-1 HA subtypes.

## Materials and Methods

### Molecular Cloning and Expression

The genes encoding wild-type HA and NA proteins of H1 NC99 (A/New Caledonia/30/1999 (H1N1)), H1 CA 09 (A/California/4/2009 (H1N1)), H2 SING 57 (A/Singapore/1/57 (H2N2)), H5 IND 05 (A/Indonesia/05/05 (H5N1)), and H9 HK 99 (A/Hong Kong/1074/99 (H9N2)), H1 stabilized stem (SS) H1 NC 99 SS, HIV gp120 control protein, and monoclonal Antibodies CR6261, CR8020, FI6v3, and F10 were synthesized^21^. The remaining HA SS probes were constructed by overlapping PCR. Genes encoding these proteins were cloned into a CMVR plasmid backbone for mammalian cell expression^21,22^. Δstem mutant probe with two point mutations, Ile45Arg/Thr49Arg (Arg point mutations in HA2; H3 numbering), which prevent binding of bNAb like CR6261 or F10 at the conserved H1 stem epitope were generated using site directed mutagenesis (QuikChange II Site-Directed Mutagenesis Kit; Agilent Technologies, CA, USA). Plasmids encoding these proteins were transfected into 293 F cells (a human embryonic kidney cell line) and supernatants were harvested 72–96 hrs after transfection. HA trimers and stabilized stem proteins were purified as previously described^21,23^. IgG Antibodies were purified using a Protein G affinity column (GE Healthcare) as described by the manufacturer.

### Characterization of HA stabilized stem

FPLC purified HA SS proteins (Supplementary Fig. 1) were characterized by ELISA using human monoclonal Antibodies CR6261, CR8020 and FI6v3^6,12,24^. H1 HA NC99 and HIV gp120 proteins served as positive and negative controls, respectively. Ab binding was detected by peroxidase-conjugated goat anti-human IgG (Rockland Immunochemicals Inc., PA, USA). Dose-response curve was plotted using absorbance reading at Y-axis and antibody dilutions on X-axis. Endpoint dilutions were determined from non-linear fit dose-response curves using a detection limit of four-fold (4 X) above background absorbance (washing buffer alone)^12^. The HA NC 99 SS was further characterized by ELISA and pseudotype neutralization competition assays. Presence or absence of the stem epitope bound by CR6261 on each of the various engineered proteins was measured by the ability of a given protein to compete the antibody CR6261 away from either substrate protein, in an ELISA, or pseudotyped virus, in a neutralization assay. Briefly, ELISA binding competition was performed by incubating CR6261 with the competitor protein (H1 NC 99, H1 NC 99 Δstem, H1 NC 99 SS, H1 NC 99 SS Δstem or gp120) at a concentration of 10 μg/ml prior to addition to plates pre-coated with H1 NC 99 (200 ng/well) and blocked with 5% skim milk^21,22^. Neutralization competition was performed as previously described^21,25^ in which serially diluted CR6261 (starting from 10 µg/mL) was incubated in the presence of a constant amount of H1 NC 99, H1 NC 99 SS, their respective Δstem Ile45Arg/Thr49Arg probes, or gp120 control (10 µg/mL) for 1 hr at RT before addition to pseudotyped recombinant HA/NA lentiviruses. The virus/antibody/competitor mixture was then applied to 293 A cells, which where incubated overnight; media was changed after 12–14 hours; and luciferase activity was measured 48 hours later as described^21,25^.

### Human Sera Collection

In this study, we used archived serum samples that were collected from healthy adult donors (United States residents) between 2004 and 2010 in six Phase I vaccine clinical trials evaluating influenza, Ebola or SARS DNA vaccines. The samples were collected after approval from relevant institutional research ethics committees and using informed consent signed by the enrolled patients. The studies were conducted at the NIH Clinical Center by the NIAID Vaccine Research Center, National Institutes of Health (Bethesda, MD) (Clinicaltrials.gov identifiers: NCT00973895, NCT00489931, NCT00776711, NCT00099463, NCT01086657, NCT00072605). Sera samples assessed in this study were collected at baseline, prior to administration of any vaccine. All protocols were approved by the NIAID Intramural Institutional Review Board and U.S. Department of Health and Human Services human experimental guidelines for conducting clinical research were followed.

### ELISA Binding and mAb competition assays

Human sera samples (n = 202) were first analyzed for their ability to bind SS probes from different subtypes (H1N1 A/New Caledonia/20/1999 (H1 NC 99) SS (seasonal), H1N1 A/California/04/2009 (H1 CA 09) SS (pandemic), H2N2 A/Singapore/1/57 H2 (SING 57) SS, H5N1 A/Indonesia/5/2005 (H5 IND 05) SS, and H9N2 A/Hong Kong/1074/1999 (H9 HK 99) SS and their corresponding Δstem (Ile45Arg/Thr49Arg) mutants. Samples with binding end-point titers to WT SS, six-fold or higher than that to Δstem probe were considered positive for the presence of antibodies targeting broadly neutralizing epitopes. Samples reactive to four out of five (4/5) probes were considered cross-reactive and were analyzed further by competition assay against F10-ScFv bNAb. Briefly, sera were diluted in blocking buffer (5% skim milk) containing constant amount (20 μg/ml) of competitor single-chain Fv (scFv) F10 or control anti-HIV scFv CH31^26^. Mixtures were then added to ELISA plates coated with H1 NC 99 SS (200 ng/well), incubated for 1 hr at RT, washed, and detected with anti-human Fc-specific secondary conjugated with HRP. Samples were considered positive for stem-epitope antibodies if they were competed by F10 but not by CH31 scFv with statistical significance at 95% confidence interval using a two-tailed t-test.

### Cross-reactive B cells flow cytometry analysis

In order to assess HA- directed B cell binding and cross-reactivity, peripheral blood mononuclear cells (PBMC’s) from samples with different stem binding based on ELISA assay (n = 18) were further analyzed by flow cytometry. Three groups were selected for the analysis: negative for stem antibodies (n = 5), reactive to one stem only (n = 4), and samples with cross-reactive stem binding (n = 9). Cryopreserved PBMC’s were thawed in R-10 media containing 50 U/mL of Universal Nuclease (ThermoFisher). Cells were washed and resuspended in PBS for staining with UV-Blue viability dye (ThermoFisher) for 20 minutes at room temperature. After washing, surface staining was performed using antibodies against IgM (G20-127, BD Biosciences), IgG (G18-145, BD Biosciences), CD8 (RPA-T8, BioLegend), CD3 (OKT3, BioLegend), CD56 (HCD56, BioLegend), CD14 (M5E2, BioLegend), CD19 (J3-119, Beckman Coulter), CD27 (O323, BioLegend), CD38 (HIT2, BioLegend), and HA probes^27–29^. H1 NC 99 and H5 INDO 05 HAs were expressed and biotinylated followed by fluorochrome labeling as previously described^29^. Stained cells were run on a BD LSRFortessa X-50 and data analysis was performed using FlowJo (TreeStar). The gating strategy is demonstrated in supplemental Fig. 2. Statistical significance at 95% confidence interval was done using two-tailed t-test.

### Neutralization assay

Cross-reactive samples (bound to 4/5 probes) based on ELISA assay were further assessed for neutralization of pseudotyped H1N1 NC 99 and H5N1 INDO 05 lentiviruses by neutralization competition as previously reported^21,25^. Briefly, serially diluted serum samples (50- to 200- fold) were incubated with or without WT and Δstem HA competitor proteins at 10 µg/mL (H1 NC 99 in the case of H1N1 neutralization and H5 INDO 05 for H5N1 neutralization) for 1 hr at RT. Pseudovirus was added and the mixtures were incubated for 30 min before applying them to 293 A cells. For certain sera samples that were found to neutralize virus pseudotyped with H1N1 NC 1999 in the presence of WT HA, the assay was repeated with serum preabsorbed with 293 F cells expressing the full-length (transmembrane) NC 99 Δstem protein, to remove head-dependent neutralization activity, as previously reported^21,25^. Samples were considered positive for stem dependent neutralization if the difference in neutralization competition between HA and HA Δstem was ≥20%.

## Results

### Characterization of HA Stabilized Stem Probes

**Figure 1:**
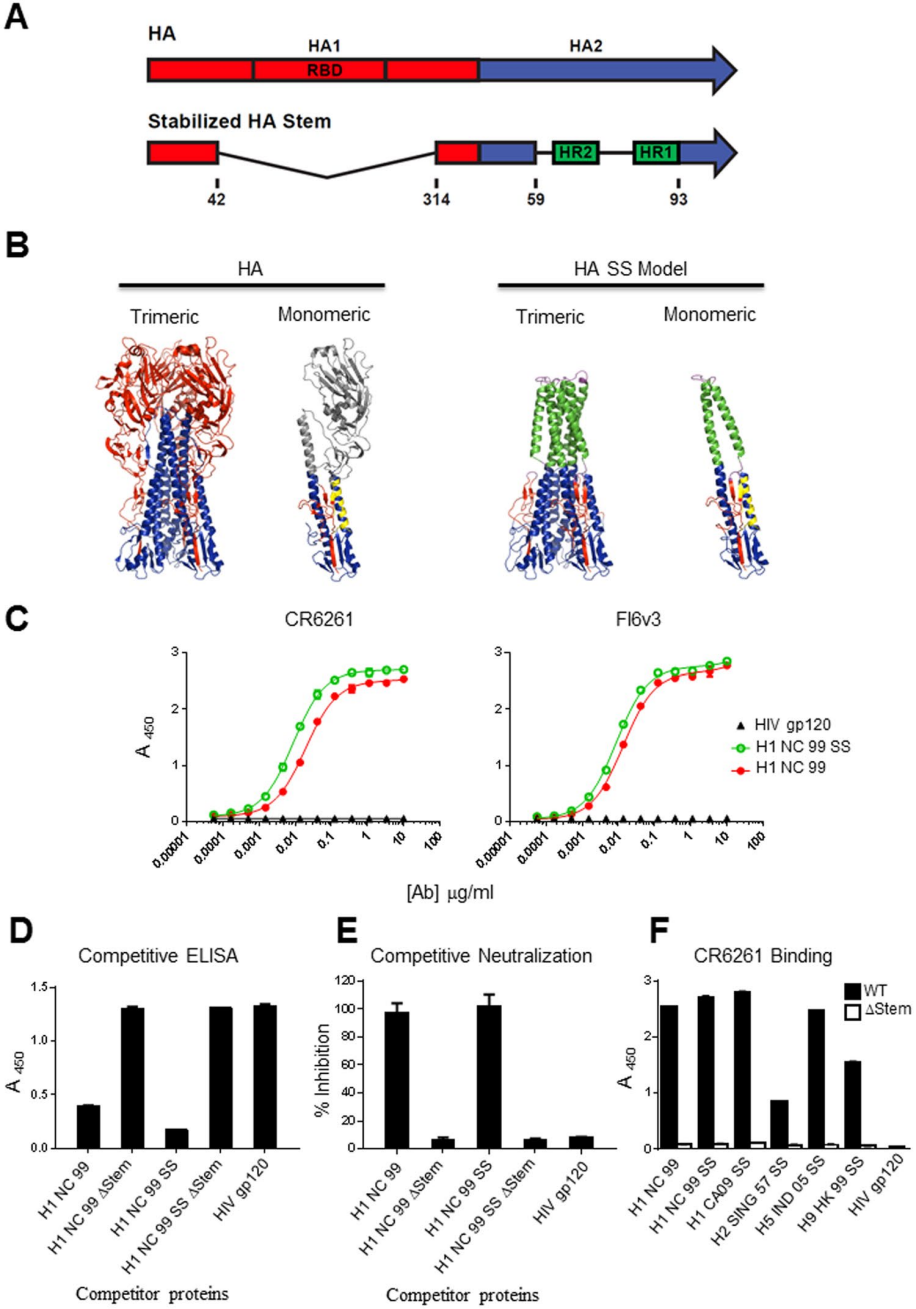
Design and characterization of HA SS protein. (**A**) Schematic of the HA SS (bottom) in comparison to HA (top). HA SS was constructed by inserting a GWG linker between residues 42 and 314 of HA1 (red) RBD head, a gp41 post-fusion trimerization motif (green) inserted in place of residues 59 through 93 of HA2 (blue), a GG linker between HA2 and the gp41 HR2 helix and an NGTGGGSG linker between the two gp41 helices. (**B**) Trimeric and monomeric representation of HA (PDB entry 1RU7) in comparison to the HA SS model. Coloring is respective to panel (A), with the monomeric representation also illustrating the CR6261 epitope as yellow and HA residues which are omitted in the stabilized HA stem as grey. (**C**) Binding of two bnAb, CR6261 and FI6v3, to WT and SS HA by ELISA. (**D**) Competition of CR6261 ELISA binding, and (**E**) pseudotyped neutralization with WT and Δstem (Ile54Arg/Thr49Arg HA2 mutations) HA and HA SS as competitors. HIV gp120 serves as a control. (**F**) CR6261 (10 µg/mL) ELISA reactivity to WT and ∆stem HA SS probes of different subtypes/strains. H1 HA NC99 and HIV gp120 serve as controls. Error bars represent standard deviation of averaged points in each panel.

To screen for antibodies targeting stem epitope of group 1 HA, we utilized a set of previously described^12^ HA SS probe constructs that lack the HA head domain while maintaining the stem region stabilized in the pre-fusion conformation (Fig. 1A,B). The probes were expressed as trimers (Supplemental Fig. 1) that preserved key structural elements as confirmed by crystal structures in complex with stem-specific monoclonal antibodies^12^. A version of the HA SS probe referred to as “Δstem” was also generated by introducing HA2 mutations I45R and T49R (H3 numbering) to block recognition of group 1, HA stem-directed bNAbs.

The initial HA SS probe derived from H1 NC 99 HA bound two previously defined stem-directed bNAbs, CR6261 (Group1 HA specific^24^) and FI6v3 (Group1 and 2 HA^6^) with affinities similar to soluble HA trimers (Fig. 1C). The integrity of the SS probe was further confirmed by ELISA and neutralization competition assays. Similar to wild-type (WT) trimeric ectodomain HA, the HA SS probe competed CR6261 reactivity to H1 NC 99 trimeric ectodomain HA (Fig. 1D) as well as CR6261 neutralization of an H1N1 NC 99 pseudovirus (Fig. 1E). In contrast, the Δstem version of HA and HA SS probes and the HIV gp120 control construct failed to inhibit CR6261 binding and neutralization activities.

Based on the same design as the H1 NC 99 HA SS probe, we generated four other probes representing pandemic H1 CA 09, H2 SING 57, H5 INDO 05, and H9 HK 99. The affinities of these probes for the CR6261 and FI6v3 were comparable to their respective HA ectodomains as determined by ELISA (Supplementary Table 1). For each, the respective Δstem mutant prevented binding by CR6261 (Fig. 1F).

### Prevalence of Group 1 HA Stem Cross-Reactive Antibodies

**Figure 2:**
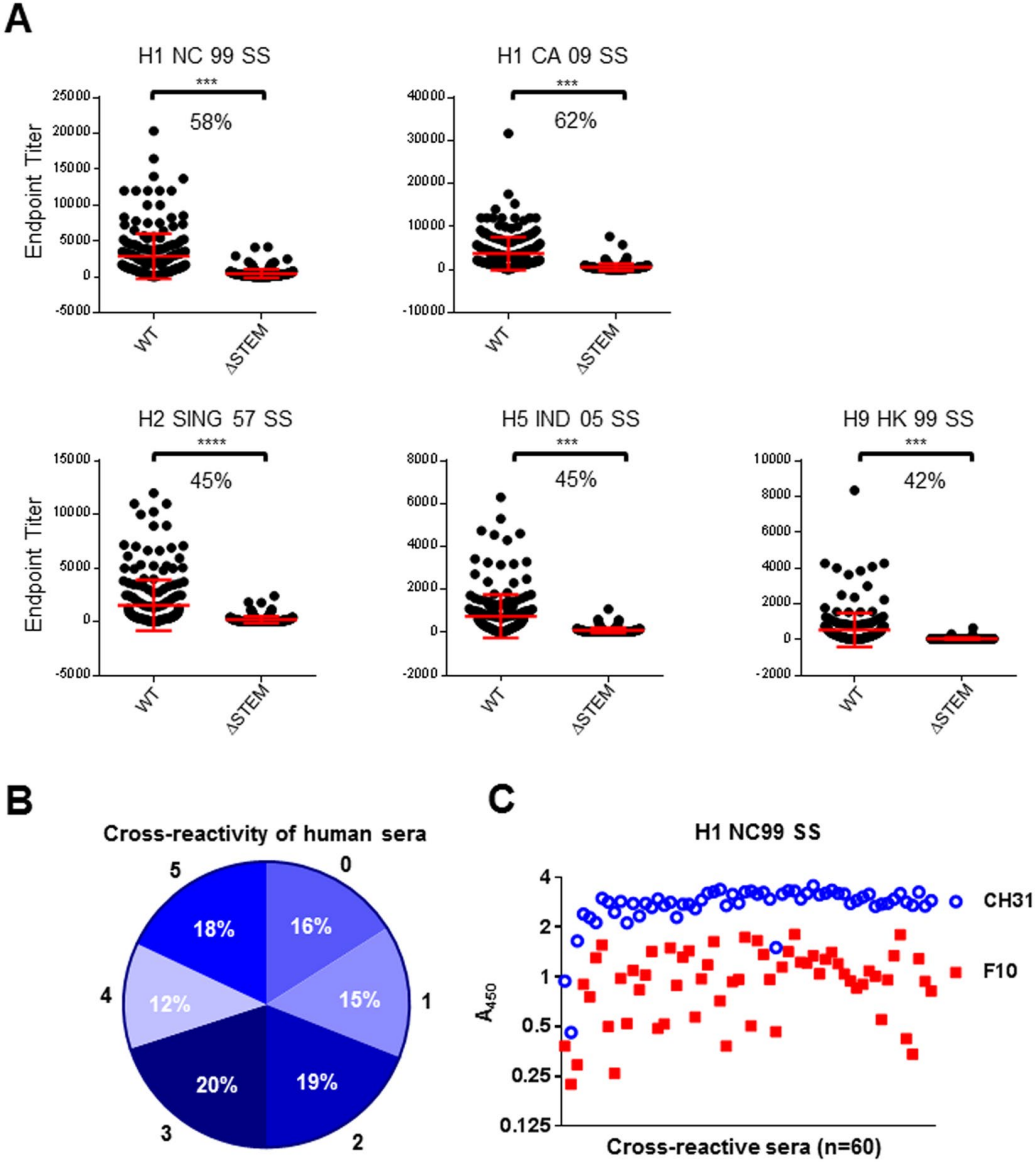
Sera reactivity to HA SS probes. (**A**) Analysis of sera binding to WT HA SS versus ∆stem probes of H1 (seasonal and pandemic), H2, H5 and H9 subtypes. Samples are considered positive for the presence of stem-directed antibodies if endpoint titers between binding to WT and ∆stem probes are greater than 6-fold. Percentages of positive samples are indicated for each subtype. Error bars (red) correspond to standard Mean plus SD. (**B**) Cross-reactivity of sera to multiple SS probes. Percentage of samples that bound to 0–5 probe(s) is shown (n = 202). (**C**) Cross-reactive sera binding-competition with F10 ScFv. Sera that reacted with 4 out of 5 probes (n = 60) were tested from stem specific antibodies in an ELISA with F10 ScFv as competitor. CH31 ScFv, HIV specific antibody, served as control.

Sera used in this study were collected at single time point from 202 random, healthy human subjects (20–65 years old) at single time points between November-2004 and May-2010. All sera samples were initially screened for HA stem antibodies by assessing their reactivity to WT HA SS versus Δstem HA SS probes (Fig. 2A). Samples with ELISA-endpoint titers 6-fold or greater to WT SS probes than to Δstem SS probes were considered positive for stem-epitope-specific antibodies. Using this criterion, 84% of the samples exhibited differential binding between WT and Δstem HA SS probes for at least one probe. About 60% of the samples had stem-epitope specific antibodies to H1 HA subtype probes (seasonal or pandemic), and between 42% to 45% were reactive to non-circulating H2, H5 and H9 subtypes probes (Fig. 2A). When analyzed for reactivity breadth, 30% of the samples tested (60 out of 202) were cross-reactive to at least 4 of the 5 probes (Fig. 2B). Moreover, an ELISA competition assay showed that the binding of these same 60 cross-reactive samples to the H1 HA SS probe was competed by F10-ScFv but not by the CH31-ScFv control antibody, confirming the presence of anti-stem epitope antibodies (Fig. 2C).

### Frequency of Cross-Reactive Memory B Cells

**Figure 3:**
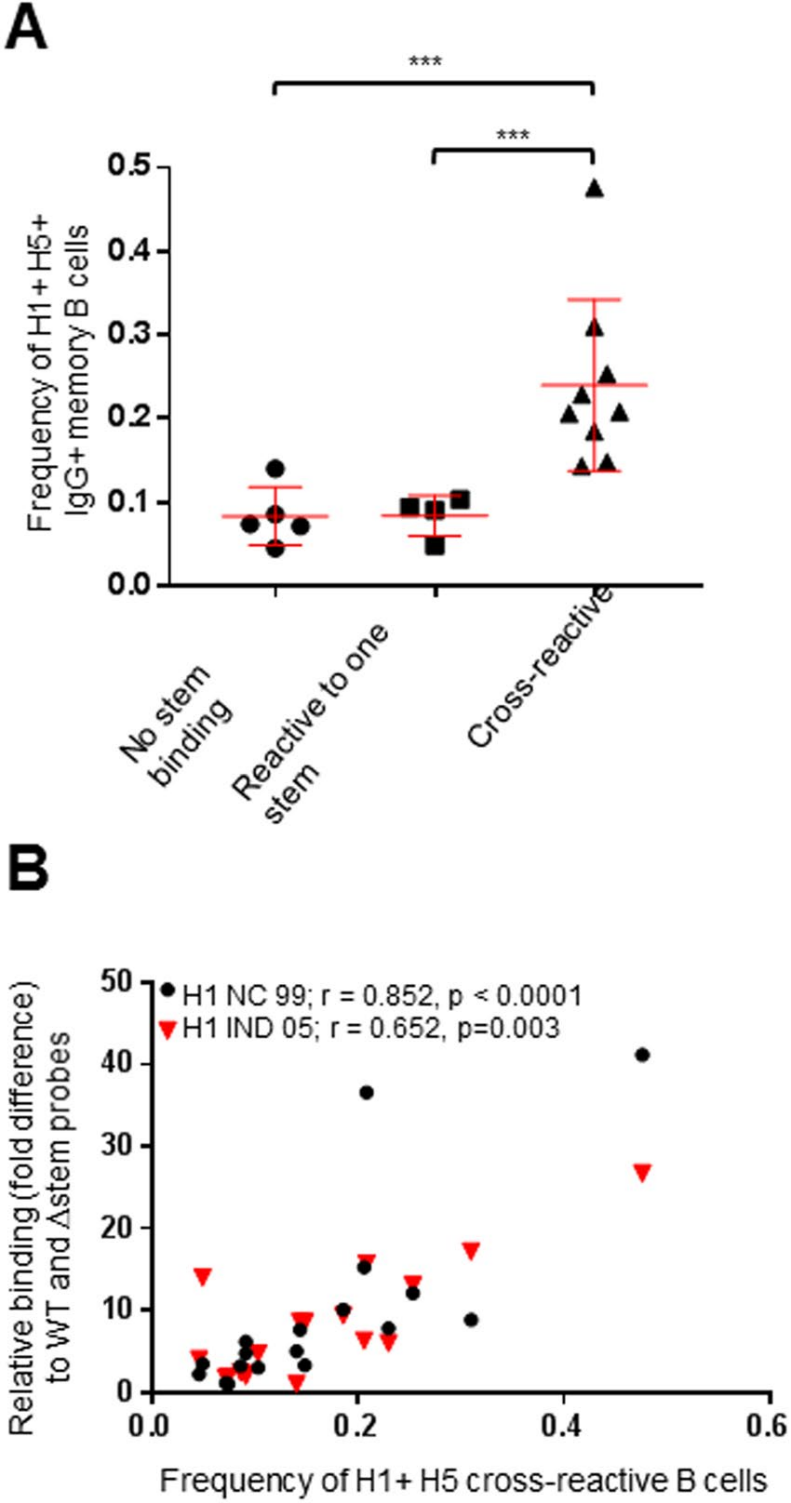
Correlation of IgG + memory B cells cross-reactivity and sera stem binding. (**A**) Frequencies of HA-specific memory B cell populations in three sera stem-binding groups: negative for stem antibodies, reactive to one stem probe only, and cross-reactive sera which binds 4/5 stem probs. Error bars (red) correspond to standard mean plus SD. Groups were compared using unpaired t-test. (**B**) Correlation between frequency of cross-reactive B cells and relative sera binding (fold difference) to WT and Δstem probes.

To determine if the presence of cross-reactive stem-specific serum antibodies correlated with cross-reactive memory B cells, we evaluated memory B cell (CD19^+^CD38^+^CD27^+^IgG^+^IgM^−^) response in 18 peripheral blood mononuclear cells (PBMCs) samples by flow cytometry using two HA probes: H1 NC 99 and H5 INDO 05 (Supplementary Fig. 2). The mean frequency of cross-reactive H1^+^H5^+^ memory B cells was statistically higher in subjects whose sera reacted to at least four different HA SS probes in ELISA (average of 0.24%) than in subjects whose sera minimally reacted with only one (average of 0.084%) or zero (average of 0.083%) probes (p = 0.003, p = 0.001, respectively) (Fig. 3A). Furthermore, the frequency of H1^+^H5^+^ cross-reactive B cells was observed to correlate strongly with the relative magnitude of serum HA stem antibody binding (defined as the fold difference reactivity between WT and Δstem HA SS probes) (Fig. 3B).

### Stem-Specific Neutralization of H1N1 and H5N1 Viruses

**Figure 4:**
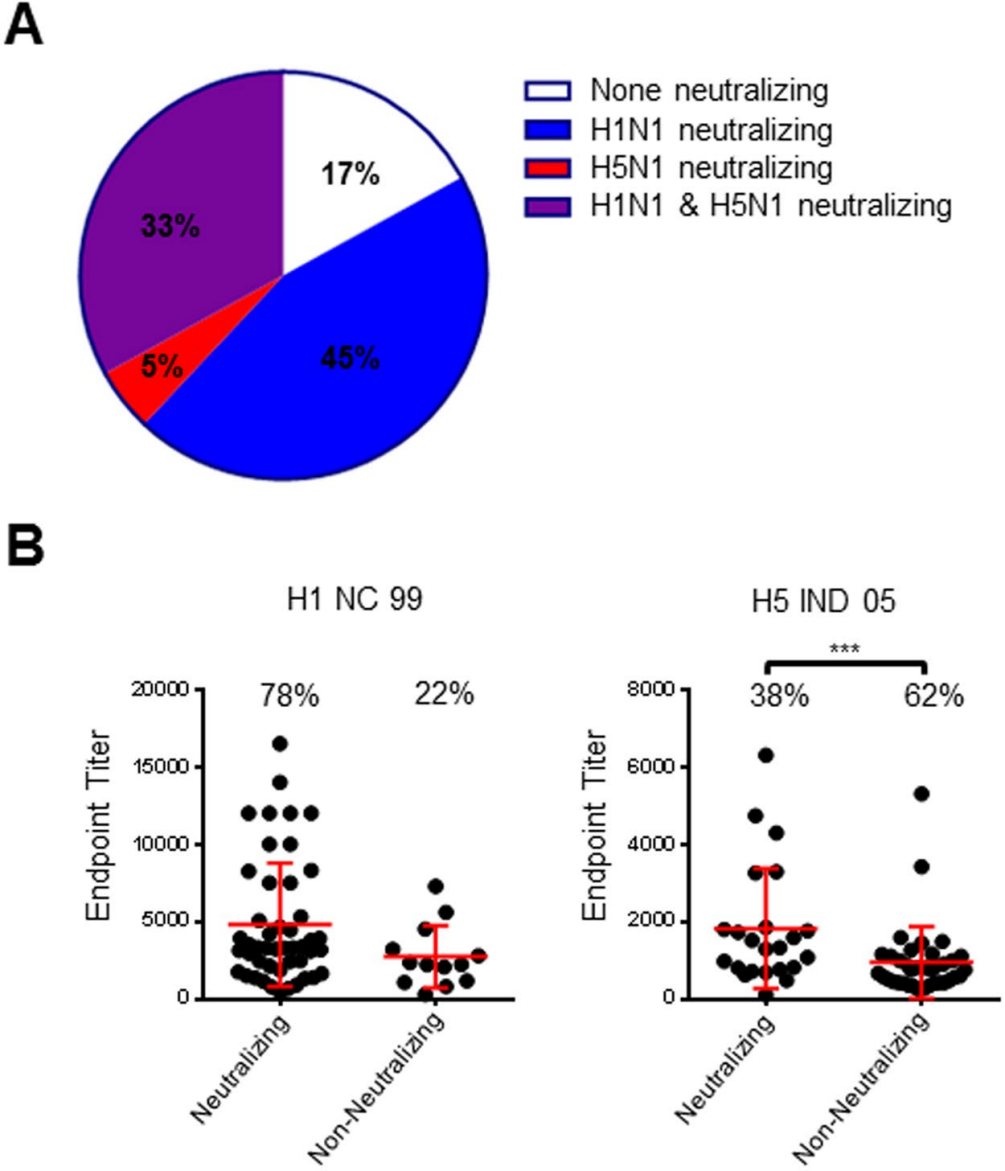
Stem-epitope dependent neutralization of H1N1 and H5N1 pseudoviruses. (**A**) Neutralization breadth of cross-reactive sera. Sera samples that bind to 4 or more HA SS probes were assayed for stem dependent neutralization of pseudotyped lentiviruses, H1 NC 99 and H5 IND 05, by neutralization competition assay. Samples with neutralization activity that is competed by WT but not Δstem HA is considered positive for stem directed nAb. The percentage of samples which neutralize H1 NC 99, H5 IND 05, both, or neither via the conserved stem epitope is shown. (**B**) Correspondence of sera neutralization activity to antibody titers. H1N1 (left) and H5N1 (right) neutralizing and non-neutralizing samples corresponding to respective ELISA endpoint titers against HA SS probes. Error bars (red) correspond to standard Mean plus SD.

Next, we evaluated the neutralization breadth of the 60 broadly cross-reactive sera samples by measuring sera neutralization of H1N1 NC 99 and H5N1 IND 05 pseudoviruses. The pseudotyped-lentivirus system was chosen due to its sensitivity, enabling the detection of neutralizing antibodies even at low titers. Samples were considered positive for stem dependent neutralization if the difference in neutralization competition between HA and HA Δstem was ≥20%. (Supplementary Fig. 3). With these criteria, 47 out of 60 samples (78%) were positive for stem-epitope dependent neutralization of H1N1 pseudovirus (Fig. 4A). Thirty-three percent of cross-reactive samples had neutralizing activity to both subtypes, and only 5% were strictly H5N1 neutralizing (Fig. 4A). In total, one third of the samples designated as cross-reactive by ELISA had significant neutralization breadth against both H1N1 and H5N1 pseudoviruses that was dependent on HA stem epitope-reactive antibodies (Fig. 4A). H1N1 neutralization was independent of stem-directed antibody titers in the sera, as we saw no significant difference in ELISA endpoint titers between neutralizing and non-neutralizing samples (Fig. 4B). However, H5N1 neutralization correlated to antibody ELISA endpoint titer against the H5 INDO 05 HA SS probe (Fig. 4B).

### Determinants of Cross-Reactive Antibodies

**Figure 5:**
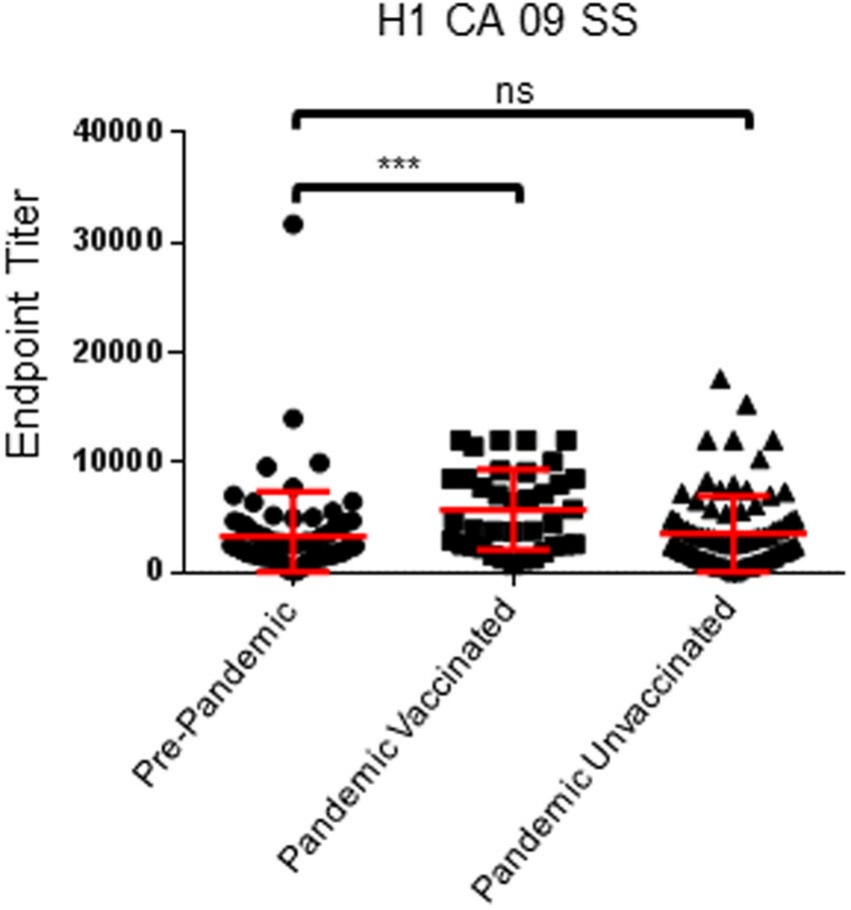
Correspondence between pandemic H1N1 exposure and anti-HA stem antibody titer in human sera. Reactivity to CA 09 HA SS of serum samples collected before and after (vaccinated and unvaccinated) the 2009 H1N1 pandemic. Error bars (red) correspond to standard Mean plus SD.

Analysis of subjects’ traits and the presence of cross-reactive stem antibodies revealed no association with gender or race. Remarkably, sera collected either prior to the 2009 pandemic or afterwards (after January 1, 2010^30–32^) showed no differences in cross-reactivity or the ability to neutralize either H1N1 or H5N1 pseudovirus (Table 1). We also observed no difference in the profiles of cross-reactive sera from vaccinated and non-vaccinated samples collected after the H1N1 pandemic emergence (Table 1). However, there was a statistically significant boost in specific stem antibody titers to H1 CA 09 HA SS probe in subjects that received pandemic H1N1 influenza vaccine compared to those that did not receive the vaccine within the same time period (Fig. 5). We also noted a higher rate of cross-reactive antibodies in the older population, especially those born within or before the period of H2N2 pandemic^33^ (Table 1). While approximately 23% of the subjects less than 30 years of age were positive for cross-reactive antibodies, the percentage increased by 1.75- and 2.40-fold in subjects between the ages 40–49 and those over 50 years of age, respectively (Table 1). The higher rate of cross-reactive antibodies in the older population was consistent with the higher neutralizing activity against of both H1N1 and H5N1 pseudoviruses. A 3–5-fold increase in H5N1 neutralization was noted in subjects between the ages 40–49 and those over 50, respectively, compared to those under the age of 30.

**Table 1:** Determinants of cross-reactive antibodies.

	N	Subjects with Stem Cross-Reactivity^a^	Subjects with Stem Cross-Reactivity and H1 Stem Neutralization	Subjects with Stem Cross-Reactivity and H5 Stem Neutralization
Age				
<30	82	19 (23%)	13 (16%)	4 (05%)
30–39	54	10 (19%)	8 (15%)	6 (11%)
40–49	35	14 (40%)	12 (34%)	5 (14%)
>50	31	17 (55%)	14 (45%)	8 (26%)
Date of Blood Draw				
Pre-2009 pandemic	79	18 (23%)	14 (18%)	6 (08%)
Post-2010^b^	63	21 (33%)	21 (29%)	8 (13%)
Pandemic Vaccination (Post Pandemic Samples^c^)				
Not Vaccinated	86	32 (37%)	25 (78%)	11 (34%)
Vaccinated	38	9 (24%)	8 (89%)	5 (55%)

^a^Samples considered to have HA stem cross-reactivity had at least a 6-fold differential binding between HA SS wt and Δstem probes for 4 of 5 probes.

^b^Samples collected after the second massive wave of the 2009 pandemic (starting from January 1^st^, 2010).

^c^Samples collected after the initial diagnosis of pandemic cases on April 14^th^, 2009.

## Discussion

An ideal influenza vaccine would trigger the immune system to recognize and target highly-conserved and vulnerable structures on the virus, such as the supersite for bNAbs on the HA stem domain^34^. Although several bNAbs that target this site have been isolated^3–7^, the profile of these antibodies in terms of their quality, quantity, specificity, and functionality in the general human population has not been thoroughly assessed. Here we provide a new serological approach for assessing the frequency, magnitude, breadth and specificity of antibodies in human sera directed to broadly neutralizing HA stem-epitope.

Our analyses improve upon other published papers^8,14–20^ in that we analyzed higher number of samples, we used structurally defined stem-only HA SS probes to detect stem-directed antibodies paired with Δstem probes to confirm the epitope targeted, and we assayed antibody binding and neutralizing breadth on individual serum samples, rather than pooled sera. The HA SS probes allow definitive measurement of stem antibodies without the potential interference of anti-HA head antibodies, while Δstem probes measure and allow us to account for antibodies targeting the specific epitope encompassing the supersite for bNAbs. Furthermore, by utilizing HA SS probes for several HA subtypes (seasonal and pandemic H1, H2, H5, and H9) we were able to directly assess the breadth of sera reactivity to all four group 1 HA subtypes known to have infected humans. In addition, we demonstrated a correlation between HA stem-specific antibody binding and neutralization with the presence of cross-reactive HA-specific B cells that have been shown in other studies to express immunoglobulins that target the HA stem^27,34^.

Analyses of sera samples with HA SS probes indicated wide prevalence of stem antibodies, as only 16% of subjects lacked bNAb-epitope-specific reactivity. As expected, reactivity to the H1 HA subtypes, both seasonal and pandemic, was commonly observed (~60%). However, 42%–45% of samples also reacted to the stem-epitope of the non-circulating subtypes (H2, H5, and H9). Furthermore, 30% (n = 60) of the samples harbored reactivity to at least 4 out of 5 probes. Our findings suggest a much higher prevalence of group 1 HA stem epitope directed antibodies in the general population (84%) than previously reported values of up to 30% in unvaccinated subjects^14^. BNAbs recognizing group 1 HA stem have been shown to arise predominantly (>60 %) from the IGHV1-69 v-gene^28,29^. Moreover, antibodies containing the VH1-69 v-gene have been observed to be expressed at high levels of up to 11%^35^. Finally, germline-reverted, VH1-69-containing, HA stem-reactive bNAbs have been shown to recognize group 1 HA stem antigens and require minimal levels of somatic hypermutation for maturation^11^. Therefore, the human immune system has evolved the capacity to generate stem-directed influenza antibodies in the large majority of individuals. The difference in the reported seroprevalence rate of stem-directed antibodies in this study compared to others could be referred to the difference in the used methodology and type of analysis. Firstly, we used multiple subtypes to screen for stem antibodies, which enables better detection coverage, and secondly, we specifically screened for epitope-specific response by utilizing WT and Δstem probes, while defining a cutoff value of end-point-binding-titer fold difference between the two probes.

We also tested whether the 60 most cross-reactive samples could neutralize virus. Most neutralized H1N1-pseudotyped virus, and more than a third neutralized H5N1-pseudotyped virus, despite no previous exposure to this subtype. Neutralizing activity against H5N1, but not H1N1 virus, correlated with the magnitude of serum antibody binding, indicating a possible correlation between antibody level in sera and the potential for heterosubtypic immunity. Variation in the ability of stem antibodies to achieve heterosubtypic neutralization might also rely on maturation pathways of specific antibody lineages and the exposure history to prior infections or vaccinations^34^.

Because our primary interest was to understand the underlying factors leading to the elicitation of bNAb, we analyzed the traits of subjects possessing cross-reactive sera capable of recognizing at least four of the subtypes tested. We found that the incidence of cross-reactive antibodies, as well as their neutralizing activity of H1N1 and H5N1 pseudoviruses, was age dependent. This could be due to a more extensive exposure to diverse influenza strains via infection or vaccination. This includes the exposure to H2N2 subtype that circulated between 1957 and 1968^33^, as well as the swine H1N1 strain that emerged in the late 1970s^36^. Moreover, childhood imprinting by the first influenza strains encountered by a subject has been suggested to play a role in adult immune response to influenza^37^. The breadth of antibody binding in sera correlated with the frequency of cross-reactive memory B cells isolated from PBMCs. These observations support the possibility that long-lived memory B cells targeting this conserved site exist^15^ and could potentially be boosted by vaccines designed to display HA stem epitopes.

Consistent with previous reports^8,15,38^, we also observed an increase in stem-directed antibodies specific for the pandemic H1 HA in individuals vaccinated for that strain. This observation supports the hypothesis suggested by others that an HA molecule with a novel head domain presented with an HA stem domain from a previous exposure may be able to boost cross-reactive HA stem-specific antibody responses^39,40^. However, we observed no difference in the prevalence of cross-reactive antibodies in samples collected before the 2009 pandemic, and those collected after January 1^st^ of 2010 (after the second wave of infection in which a substantial percentage of the population is thought to have encountered the virus by infection or vaccination)^30–32^. Clearly, many people might not have been exposed to the virus after the second wave, whether by vaccination or infection, which partially explain our results.

Although we were looking for epitope-specific antibodies by utilizing WT and Δstem probes, our data suggest that some HA stem-specific antibodies could be subtype specific, as denoted by the lower frequency of antibody binding to non-circulating subtypes (H2, H5, and H9) compared to circulating subtypes (seasonal and pandemic H1). Furthermore, although significant number of sera samples were broadly reactive in terms of stem binding as measured with ELISA, only a subset of these (30%) were able to neutralize a divergent non-circulating group 1 virus (H5N1). These observations suggest that subtle variations in the specificity of stem-directed antibodies or structures of HA stem surfaces between subtypes may result in varying degrees of breadth. Our resluts also indicate that these antibodies could be cross-reactive but not necessarily cross-neutralizing, partially depending on their titers in the sera. Whether such cross-reactivity translates into other effector mechanisms such as ADCC and CDC requires further investigations. Importantly, these observations suggest that HA stem-based vaccination strategy might require the use of multiple components to provide broader protection, especially when it comes to cross-group neutralization such as H3 and H7.

Overall, this emphasizes the importance of evaluating reactivity against multiple HA stem subtypes to gauge the breadth of antibody cross-reactivity, especially when evaluating the immunogenicity of candidate universal influenza vaccines. It is worth noting here that stem-directed antibodies could provide protection against heterosubtypic pathogenic viruses regardless of the neutralization activity as we reported earlier in mouse and feret models^12^. This mandates the development of additional procedures to measure other activities of stem-directed antibodies, such as antibody-dependent cell-mediated cytotoxicity (ADCC) and antibody-mediated complement-dependent cytotoxicity (CDC).

Our results and those of others^14,15^ leave several questions unanswered: why do we need annual vaccination to protect from influenza infection if stem-directed antibodies are prevalent, what is the minimal titer of HA stem-specific antibody required for immunity against influenza disease, would the effectiveness of HA stem-specific antibody differ in upper versus lower respiratory tract infection, will an HA stem-based vaccine antigen provide immunity against strains within a subtype, across subtypes within a group, or across groups of influenza A?

It is worth noting that ADCC and CDC would be more prominent in the lung due to the availability of effector cells in such environment, while most of the circulating influenza viruses tend to cause upper respiratory tract infection (URTI). On the other hand, H5N1 viruses are known to cause lower respiratory tract infections (LRTI) in humans. Interestingly, a meta-analysis of studies that evaluated the serological evidence of H5N1 infections in humans indicated a seropositivity up to 2% in high risk groups and concluded that H5N1 viruses can cause mild or subclinical infections in humans^41^. Our findings in animal models indicate that stem antibodies can protect against lethal H5N1 infection although they lack measurable neutralizing activity against the virus *in vitro*, which suggest the involvement of other mechanisms such as ADCC and CDC. This suggest that stem antibodies might provide better protection from LRTI rather than URTI. Evaluating anti-stem antibodies in passive transfer experiments in human challenge studies to define whether neutralization or Fc-mediated effector functions were required for protection would be highly informative.

In summary, we developed an important tool for measuring stem-directed antibodies in human sera and illustrated how these assays will inform vaccine development. Our analyses indicates that stem-directed antibodies are prevalent in human sera across age groups, although the breadth and function of antibody’s binding and neutralization can be dependent on age and presumably the exposure history. Therefore, it may be feasible to design candidate vaccines that can effectively boost pre-existing responses with cross-protective capacity. Our analysis also shows that not all stem-directed antibodies in human sera are equally broad. Therefore, universal influenza vaccine candidates will need to be designed to activate selected precursors and may require more than one component to generate the desired breadth of protection.

## Electronic supplementary material

Supplementary Information
