# Supplementary material for: Use of Hemagglutinin Stem Probes Demonstrate Prevalence of Broadly Reactive Group 1 Influenza Antibodies in Human Sera
Source: Sci Rep. 2018 Jun 5;8:8628. doi: 10.1038/s41598-018-26538-7 (PMC5988737; doi:10.1038/s41598-018-26538-7)
Supplement: Supplementary file 1 — Supplementary Information [file 41598_2018_26538_MOESM1_ESM.pdf]

Supplementary Information

Use of Hemagglutinin Stem Probes Demonstrate Prevalence of Broadly Reactive Group 1 Influenza Antibodies in Human Sera

Hadi M. Yassine[1*], Patrick M. McTamney[2], Jeffery C. Boyington[3], Tracy J. Ruckwardt[3], Michelle C. Crank[3], Maria K. Smatti[1], Julie E. Ledgerwood[3], Barney S. Graham[3*]

1 Qatar University Biomedical Research Center, Doha, Qatar 2713.

2 Medimmune, Gaithersburg, MD 20878.

3 Vaccine Research Center, National Institute of Allergy and Infectious Diseases, Bethesda, MD 20892.

Supplementary Table 1: BnAb EC_{50} to HA and SS probes

Antigen	EC_{50} (ng/mL) CR6261	EC_{50} (ng/mL) FI6v3
Ectodomain		
H1 NC 99	19.4 ± 0.8	13.5 ± 0.7
Stabilized Stem		
H1 NC 99	8.8 ± 0.3	8.6 ± 0.8
H1 CA 09	10.6 ± 0.5	11.6 ± 0.4
H2 SING 57	308 ± 52	8.5 ± 0.5
H5 IND 05	22.0 ± 1.9	9.8 ± 0.6
H9 HK 99	581 ± 28.5	8.4 ± 0.3
H3 HK 68	$> 1 \times 10^6$	14.8 ± 0.8

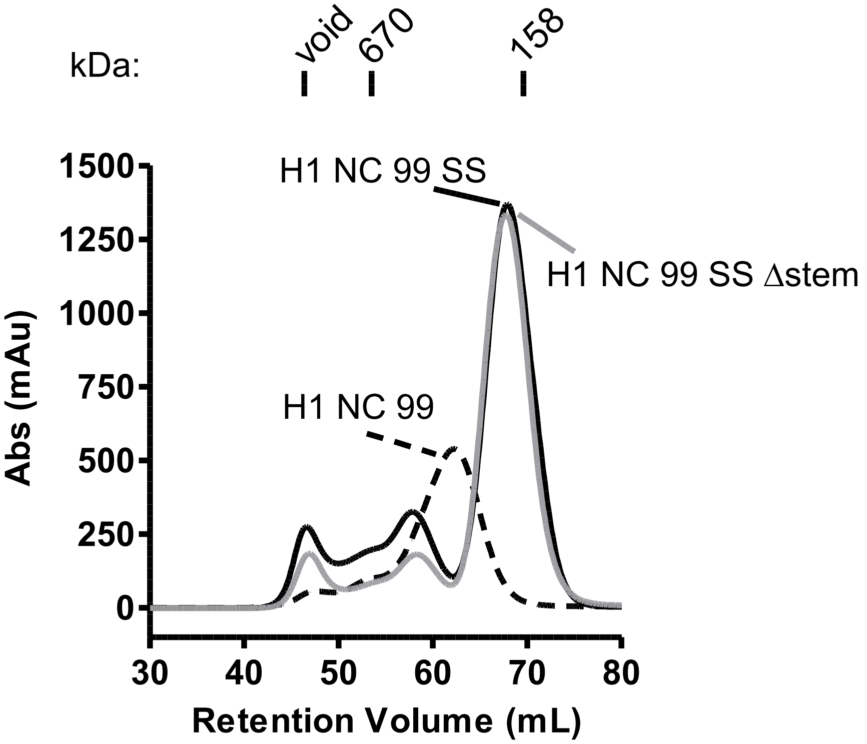

Supplementary Figure 1. Size exclusion chromatogram of HA and HA SS probes.
Proteins were first purified by Ni^{2+}-affinity chromatography followed by size exclusion chromatography (HiLoad 16/60 Superdex^{Tm} 200 pg; GE Healthcare). UV absorption (at 280 nm) showed that each protein eluted as a major single peak as a trimer with an apparent molecular weight of approximately 305, 188, and 197 kDa for trimeric HA and SS HA, and SS Δstem, respectively. Protein molecular weight standards are shown above the curves as vertical lines.

A

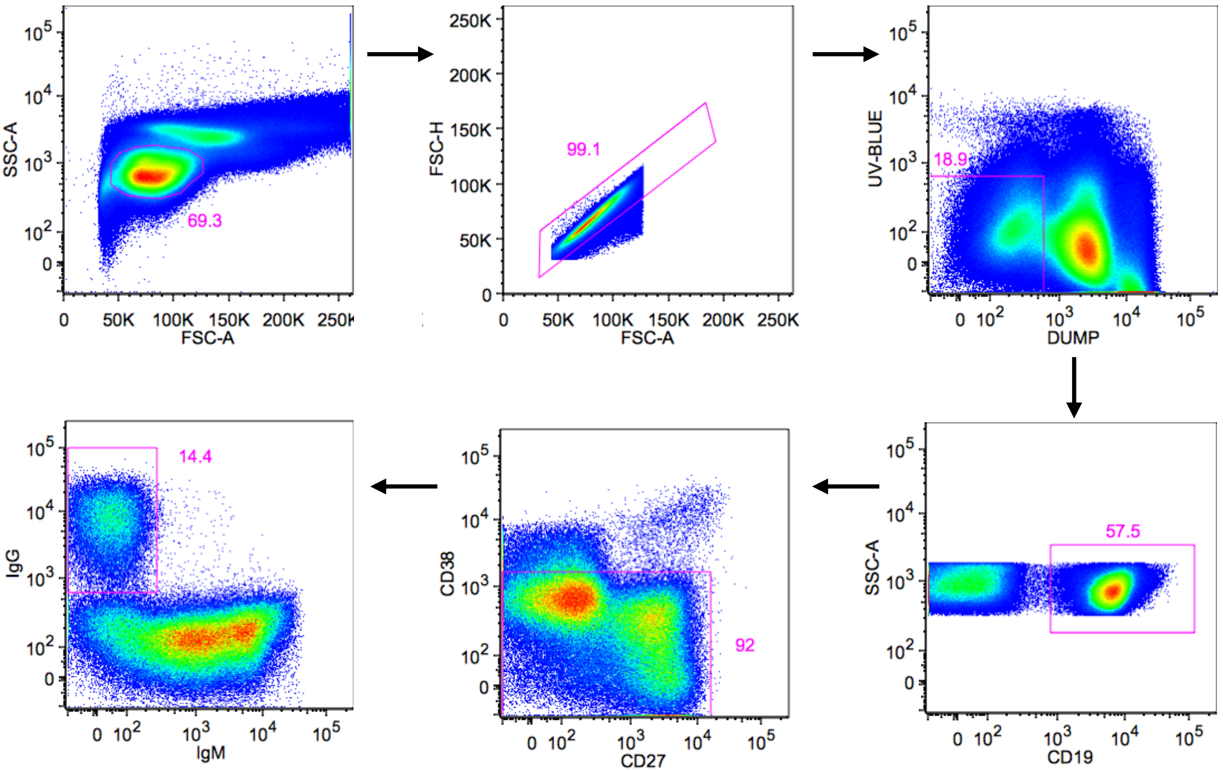

B

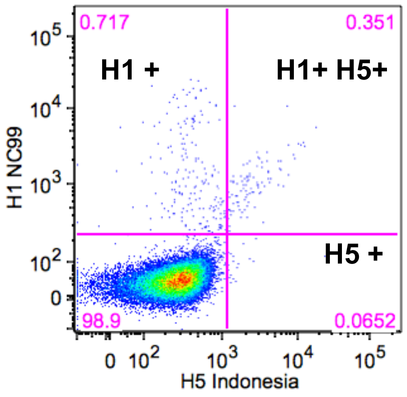

Supplementary Figure 2. Gating strategy for memory B cells from PBMCs samples
(A) IgG+ memory B cells were defined as CD3-CD8-CD14-CD56-CD19+CD38+CD27+IgG+IgM-. Dead cells were excluded using UV-Blue viability dye. **(B)** Representative staining of memory B cells with H1 and H5 probes.

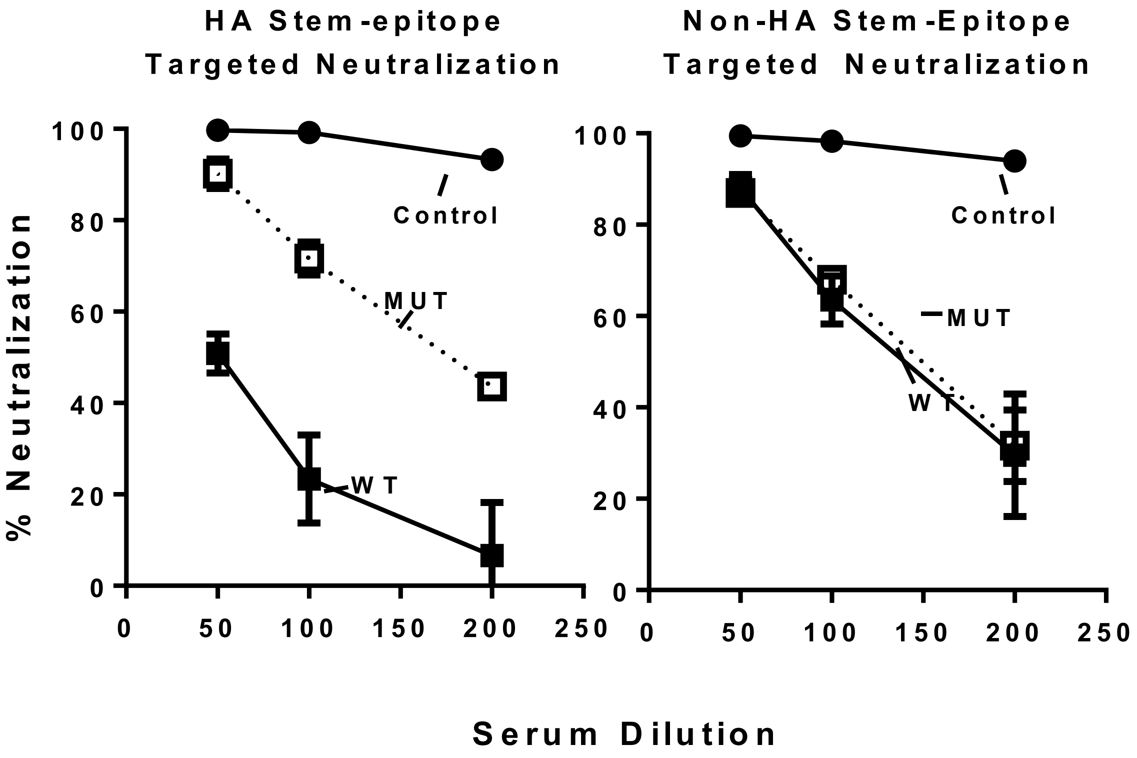

Supplementary Figure 3. Representative of stem-specific neutralizing (left) and non-neutralizing (right) sera samples against H1N1 NC 99.

Samples that bind to four or five HA SS probes (n=60) were assessed for stem-dependent neutralization of H1N1 NC 99 and H5N1 IND 05 in the presence of WT or Δstem HA as competitors. Neutralization in the absence of protein competitor serves as control. Samples were considered positive for stem dependent neutralization if the difference in neutralization competition between HA and HA Dstem was ≥20%.
